# RNA-Seq identifies genes whose proteins are transformative in the differentiation of cytotrophoblast to syncytiotrophoblast, in human primary villous and BeWo trophoblasts

**DOI:** 10.1038/s41598-018-23379-2

**Published:** 2018-03-23

**Authors:** Christopher Azar, Mark Valentine, Julie Trausch-Azar, Todd Druley, D. Michael Nelson, Alan L. Schwartz

**Affiliations:** 1 Departments of Pediatrics, Washington University School of Medicine, St. Louis, MO 63110 USA; 2 Genetics, Washington University School of Medicine, St. Louis, MO 63110 USA; 3 Obstetrics and Gynecology, Washington University School of Medicine, St. Louis, MO 63110 USA; 4 Developmental Biology, Washington University School of Medicine, St. Louis, MO 63110 USA

## Abstract

The fusion of villous cytotrophoblasts into the multinucleated syncytiotrophoblast is critical for the essential functions of the mammalian placenta. Using RNA-Seq gene expression and quantitative protein expression, we identified genes and their cognate proteins which are coordinately up- or down-regulated in two cellular models of cytotrophoblast to syncytiotrophoblast development, human primary villous and human BeWo cytotrophoblasts. These include hCGβ, TREML2, PAM, CRIP2, INHA, FLRG, SERPINF1, C17orf96, KRT17 and SAA1. These findings provide avenues for further understanding the mechanisms underlying mammalian placental synctiotrophoblast development.

## Introduction

The mammalian placenta is a specialized organ that serves essential roles in fetal growth and development throughout pregnancy including regulated delivery of nutrients to the fetus and elimination of metabolic wastes, regulated gas exchange and secretion of hormones that maintain pregnancy. Placental dysfunction underlies both short-term (e.g. fetal growth restriction) and long-term (e.g. consequences of developmental origins of adult disease) sequelae for the fetus-newborn-child-adult.

The syncytiotrophoblast of term placenta is a multinucleated, continuous layer surfacing of villi. The subjacent, proliferative villous cytotrophoblasts replenish the overlying syncytiotrophoblast through membrane–membrane fusion events. Importantly, the mechanisms that govern the transition of villous cytotrophoblasts into syncytiotrophoblast are central to understanding the physiological and pathological roles of this key maternal-fetal interface.

The fusion of cytotrophoblasts is tightly regulated, as reflected by the constancy of the 9/1 ratio of nuclei in villous syncytiotrophoblast compared to cytotrophoblasts from the first to third trimesters of pregnancy^1^. The processes that regulate villous cytotrophoblast fusion with syncytiotrophoblast are largely unknown, but pathways to multinucleated syncytia are better understood in myoblast cell-cell fusion^2^. For example, myoblast fusion in Drosophila involves a two-component process that signals through multiple pathways to effect actin cytoskeleton rearrangement with receptor-mediated membrane juxtaposition which is followed by membrane destabilization^3^. Moreover, recent studies in mammalian myoblast fusion identified key regulators of this process, including ferlin, myomaker, and myomixer^4–7^. Mediators identified to associate with cytotrophoblast fusion include syncytin-1, syncytin-2, SLC1A5, MFSD2A, galectin-1, transglutaminase 2-dependent deamidation of G3PD, and intermediate conductance Ca^2+^ activated channels^8–14^. Best studied among these are syncytin-1 and syncytin-2, which are fusogenic Env glycoproteins of human endogenous retroviral origin. Initially identified by Mi *et al*.^14^ and Blond *et al*.^9^, syncytin-1 is a member of the HERV-W family and confers the capacity for homo- and heterotypic fusion when expressed in cell lines expressing the syncytin-1 receptor SLC1A5^13^. Knockout of the murine syncytin-1 (i.e. syncytin-A) results in defective placental structure, which includes an absent labyrinth, excess unfused cytotrophoblasts, and embryonic lethality at 11.5-13.5 dpc^15^. Yet, the presence of syncytin-1 and its receptor in human cytotrophoblast cell lines is, in itself, insufficient for trophoblast fusion^16,17^. Thus, additional critical components of the fusion machinery must exist.

Villous cytotrophoblasts isolated from term human placentas undergo differentiation and fusion into syncytia during culture^18,19^. Moreover, a cloned cell line, BeWo b30^20^., was derived from the parental human trophoblast choriocarcinoma BeWo, and is responsive to signals for cell fusion. Indeed, we showed that enhanced cyclic AMP signaling in the b30 clone, by exposure to forskolin or 8-bromo-cyclic AMP, markedly enhances syncytia formation and hormonal differentiation, as reflected by elevated HCG synthesis and secretion^16,20^. We herein exploit the power of these two cell models to undertake an unbiased approach to identify genes, and gene products, which are coordinately regulated, either both up or both down, in the two cell systems that reflect human cytotrophoblast fusion to syncytiotrophoblast.

## Results

### BeWo cells form syncytia upon forskolin treatment

**Figure 1:**
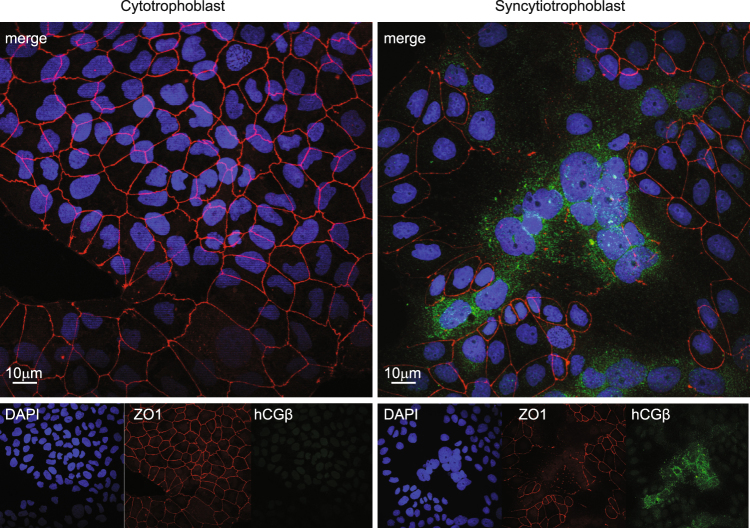
Triple-label immunofluorescence localization of BeWo cytotrophoblasts (left) and syncytiotrophoblasts (right). BeWo cells were treated with vehicle control (left) or with forskolin (right), and after 72 hours, cells were fixed, stained with DAPI (blue), anti-ZO1 (red), or anti-hCGβ (green), and imaged with an Olympus confocal microscope.

**Figure 2:**
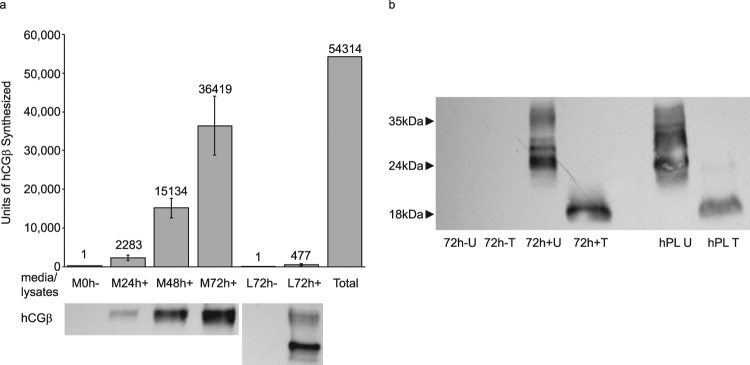
(**a**) Immunoblot analysis of hCGβ expression in media (M) and lysates (L) of BeWo cells treated with forskolin (+) or vehicle control (−) as described in Materials and Methods. The last column shows the total (media + lysate) increase in hCGβ expression compared to vehicle-treated control. (Column values are adjusted based on sample volumes analyzed). (**b**) Immunoblot analysis of hCGβ expression in BeWo cells treated with forskolin (+) or vehicle control (−) as well as human villous placental extract (hPL) with (T) and without (U) deglycosylation treatment. In both samples, deglycosylation produced the 18 kDa band predicted by the amino acid sequence of hCGβ.

BeWo cells grow in culture as cytotrophoblasts. Incubation with 100 µM forskolin for 72 h induces marked syncytia formation, as we have shown previously^20^ (Fig. 1). In the absence of forskolin, cytotrophoblasts maintain a contiguous monolayer with prominent Z01 staining at epithelial borders. No staining for hCGβ is detectable above background. In contrast, in the presence of forskolin for 72 h, there are abundant multi-nucleate syncytia, which show larger, clustered nuclei, display loss of Z01 staining, and exhibit abundant hCGβ staining within cytoplasmic vesicles, all compared to vehicle control BeWo cultures. Analysis of cell lysates and media during the 72 hour time course revealed essentially no detectable hCGβ in either cells or media in the absence of forskolin. Whereas a time-dependent accumulation of hCGβ was seen upon forskolin treatment (Fig. 2a). The total hCGβ synthesized (i.e. total media + cells) at 72 h in the presence of forskolin was ~5.4 × 10^4^–fold greater than that seen at baseline in vehicle control treated cells in the absence of forskolin. Only the 35 kDa fully glycosylated form of hCGβ was detected in media, whereas in cell lysates both the 35 kDa fully glycosylated and the 24 kDa partially-glycosylated hCGβ forms were also detected. This is likely a consequence of such a marked induction in hCGβ synthesis and processing that the glycosylation machinery within the cells was overwhelmed (Fig. 2a,b).

### RNA-Seq of BeWo cells and human placental trophoblasts

**Figure 3:**
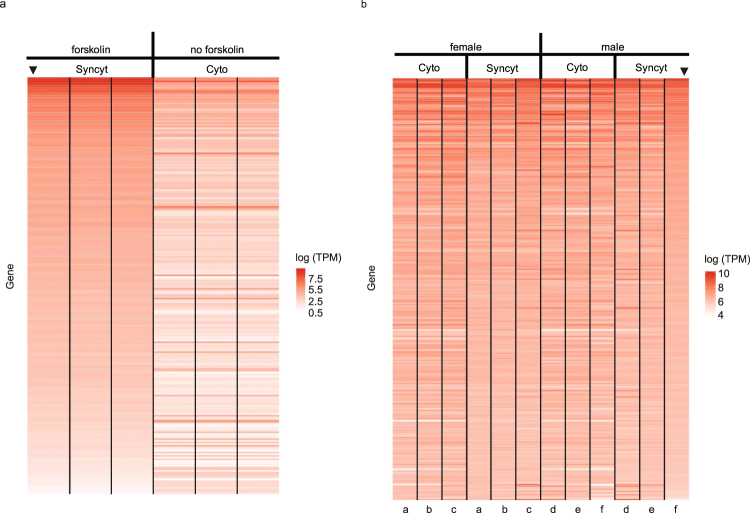
RNA-Seq gene expression heat maps of triplicate samples for BeWo cells +/− forskolin. (**a**) Three independent female (a,b,c) and three independent male (d,e,f) samples of human term villous cytotrophoblasts and the syncytiotrophoblasts that evolve *in vitro*. (**b**) In each plot, the arrowhead denotes the sample used to order the heat map. Each letter denotes the same placental sample at a given time point.

RNA-Seq was performed on triplicate cultures of BeWo cells at 0 h and 72 h in the absence of forskolin and at 24 h, 48 h, and 72 h in the presence of forskolin. As seen in Fig. 3a, there was a remarkable similarity in overall gene expression levels among the three samples at each of the time points. Moreover, there were minimal differences between the 0 h and 72 h samples in the absence of forskolin, whereas there were substantial differences among the 24 h, 48 h, and 72 h samples in the presence of forskolin, relative to the vehicle-treated samples. A principal component analysis of these data is seen in Supplemental Fig. 1a and demonstrates the time course of the forskolin effect.

RNA-Seq was also performed on three male and three female placental cell cultures harvested at 24 h when the cytotrophoblast phenotype is >98% and 72 h when the syncytiotrophoblast phenotype is the most prominent. As seen in Fig. 3b, there was minor variation in the gene expression pattern among the three male and three female samples at 24 h. Similarly, there was minor variation in the expression patterns among the three male and three female samples at 72 h. However, there were substantial differences among the six 24 h (cytotrophoblast) samples, compared to the six 72 h (syncytiotrophoblast) samples. This pattern was exactly as anticipated, with the minor differences between samples at the same time point representing the variability present in genetically distinct samples. In contrast, the much larger differences in the same sample between time points captures the different transcriptional programs in place in cytotrophoblasts and syncytiotrophoblasts. A principal component analysis of these data is seen in Supplemental Fig. 1b and demonstrates the overlap of male and female samples at 24 h and at 72 h, yet distinction between all cytotrophoblast (24 h) and all syncytiotrophoblast (72 h) samples.

**Figure 4:**
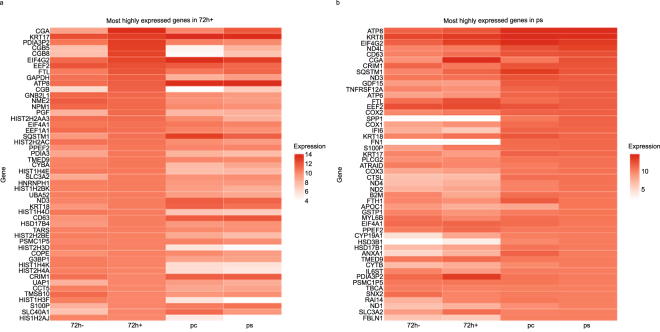
The most highly expressed genes in the BeWo cells (**a**) and human term primary villous cytotrophoblasts and their evolution to syncytiotrophoblasts *in vitro* (**b**). In each panel, the 50 most highly expressed genes in the indicated sample were selected and used to sort the heat map. The expression levels of those genes in each of the other samples is also shown.

To determine the genes differentially expressed in the BeWo cells + forskolin (i.e. syncytiotrophoblasts) vs. the BeWo cells – forskolin (i.e. cytotrophoblasts), we analyzed the 50 most highly expressed genes in BeWo cells + forskolin and compared them to BeWo cells – forskolin and to the primary cells’ syncytiotrophoblast and cytotrophoblast models. A number of the most highly expressed genes during the BeWo transition from cytotrophoblast to syncytiotrophoblast was in the HCG family, including CG α and CG β, but three other highly expressed gene groups encoded histone variants, transcriptional elongation factors, and cytoskeletal proteins (Fig. 4a; Supplemental Table 1). Importantly, primary cells transitioning from the cytotrophoblast to syncytiotrophoblast phenotype also exhibited the most highly expressed genes within the HCG family members, along with several transcriptional elongation factors. Interestingly, there were no histone variants among the top 50 most highly expressed genes between the two models of the villous trophoblast phenotypes (Fig. 4b; Supplemental Table 2). Instead, primary cells expressed several members of the cytochrome oxidase gene family. These differences between the two model systems demonstrate that there are distinct similarities, and differences, to the formation of syncytia in these two models that would be undetected without the direct comparison of choriocarcinoma-derived BeWo cells and primary human trophoblasts. Encouragingly, many of the genes in the cytotrophoblast to syncytiotrophoblast transition in both BeWo cells and primary human trophoblast cells overlap. This lends support to the use of both of these models and suggests that, despite some interesting differences, both are capturing important and shared aspects of the syncytialization process.

We next sought to rigorously characterize the pathways and gene families in our samples by using Panther^21^ to perform a pathway analysis of the changes in gene expression in the BeWo cells +/− forskolin and the primary cells that transitioned from cytotrophoblast to syncytiotrophoblast. The gene ontology terms that were significantly enriched in our genes included sex differentiation, reproduction, tissue development and amino acid transport. These pathways are consistent with our understanding of the syncytialization process and yield insights into the cellular functions that are required for this transition. A complete list of significant pathways is included in Supplemental Table 3.

**Figure 5:**
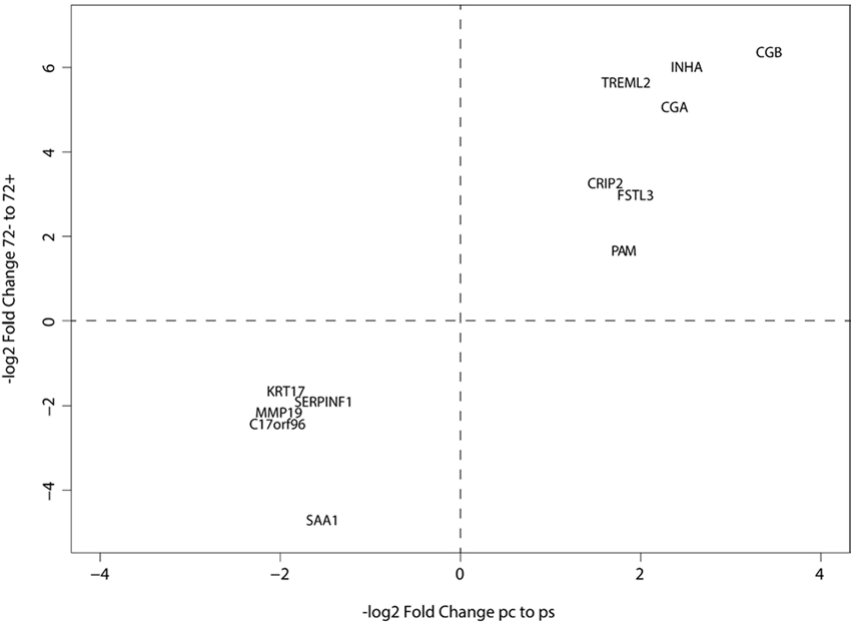
The set of genes that are differentially expressed in both cell systems. Each gene that met our fold change and expression criteria was plotted by its fold change in transformation from term human placental cytotrophoblasts to syncytiotrophoblasts on the x-axis and the fold change in BeWo cells cultured without forskolin or with forskolin for 72 h, on the y-axis. Only genes that were up-regulated in both samples or down-regulated in both samples were retained.

To best capture the genes that are critical in syncytialization, we combined our datasets into a joint analysis. To do this, we first stringently filtered each dataset independently, keeping only genes that were expressed inx ≥85th percentile for at least one-time point, while also showing greater than 2.5-fold change between the cytotrophoblast and syncytiotrophoblast. We subsequently plotted the genes as points determined on their fold change in human placenta syncytiotrophoblast/cytotrophoblast by their fold change in BeWo +/− forskolin (Fig. 5). Viewed this way, six genes were up-regulated under both conditions: HCGβ, TREML2, CRIP2, PAM, INHA and FLRG. Similarly, five genes were down-regulated under both conditions: SERPINF1, MMP19, C17orf96, KRT17 and SAA1. We also performed a parallel analysis of our RNA-Seq data using two alternative pipelines (Cuffdiff and EdgeR) as described in Methods. We found that the genes that we selected for our focused analysis were indeed found to be differentially expressed in these analyses as well (Supplemental Tables 4 and 5). We selected this list of eleven genes for further analysis.

### Protein expression in BeWo cells + /− forskolin and human placental trophoblasts

**Figure 6:**
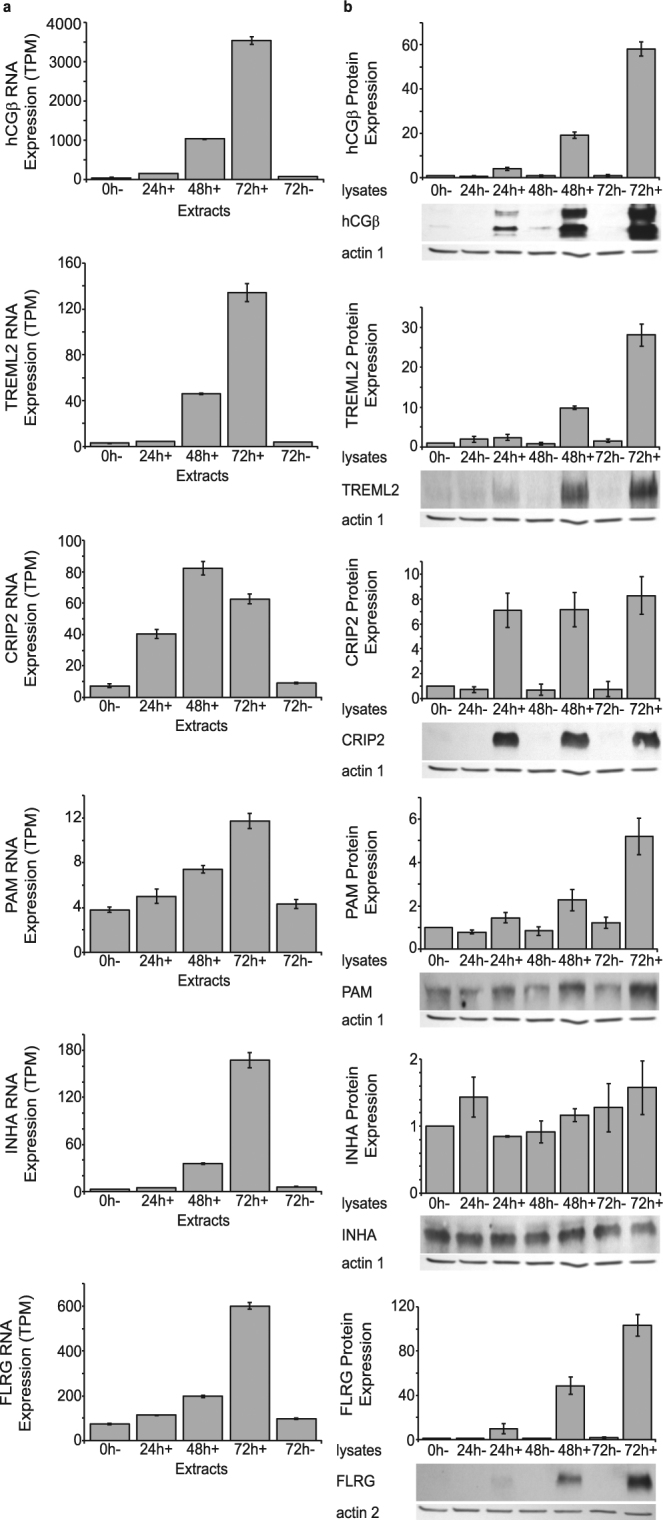
(**a**) RNA-Seq analysis of up-regulated genes of interest in vehicle treated BeWo cells (−) or BeWo cells treated with forskolin (+) for the times described in Materials and Methods. Results are expressed in transcripts per million (TPM). Each bar represents the mean + /− SEM (n = 6). (**b**) Quantitative Western blot analysis of up-regulated genes of interest in vehicle treated BeWo cells (−) or BeWo cells treated with forskolin (+) for the times described in Materials and Methods. Immunoblots were quantified and normalized to levels of actin and subsequently to the 0h- sample of each blot.

In order to determine if the changes in RNA levels (i.e. RNA-Seq expression) in BeWo cells + /− forskolin are reflected in the levels of the respective proteins, we performed quantitative Western blots of BeWo cells at 0, 24, 48 and 72 h + /− forskolin. Supplemental Table 6 lists the specific antibodies used for these analyses. As seen in Fig. 6 and Table 1 for the six genes which are up-regulated, the increase in protein expression in cell lysates (Fig. 6) correlated with the increase in RNA in five of the six genes, INHA being the exception. Of note, the fold-change likely underestimates overall protein synthesis and secretion for hCGβ, FLRG, PAM, INHA, which have secreted isoforms, as discussed above for HCGβ (Fig. 2). We also performed quantitative Western blots of human placental trophoblasts at 24 h (cytotrophoblasts) and 72 h (syncytiotrophoblasts) as samples allowed. For hCGβ and TREML2 the increase in protein expression in cell lysates correlated with the increase in RNA (Supplementary Figure 2).

**Table 1:** Summary of expression data from RNA-Seq and quantified immunoblot results shown in Figs 6 and 7. Data for 72 h vehicle control (−) or forskolin treated (+) cells are shown. Increases or decreases in expression are shown here as fractional change. Note in Fig. 5 that changes in RNA levels are expressed as -log2 fold change.

Gene	RNA Expression			Protein Expression		
**Up-Regulated**	72h−	72 h +	Fractional Change	72h−	72 h+	Fractional Change
hCGβ	75	3500	47	0.49	64	130
FLRG	99	600	6.1	1.8	118	66
CRIP2	2.2	20	9.1	1.6	74	46
TREML2	3.7	130	35	0.97	35	36
PAM	4.3	12	2.8	1.5	6.1	4.1
INHA	5.8	170	29	0.94	0.92	0.98
**Down-Regulated**						
SERPINF1	15	3.4	0.23	0.94	0.32	0.34
SAA1	11	0.50	0.05	0.57	0.26	0.46
C17orf96	9.4	2.4	0.26	0.62	0.44	0.71
KRT17	100	26	0.26	0.27	0.31	1.1
MMP19	72	25	0.35	0.38	0.57	1.5

In addition to quantitative protein analyses in BeWo cells and human plancental trophoblasts, we examined normal human placental villi using immunostaining for each of the six proteins which were up-regulated. As seen in Supplemental Fig. 3, there is robust immunostaining in the villous syncytiotrophoblasts for hCGβ, TREML2, CRIP2, PAM and FLRG.

**Figure 7:**
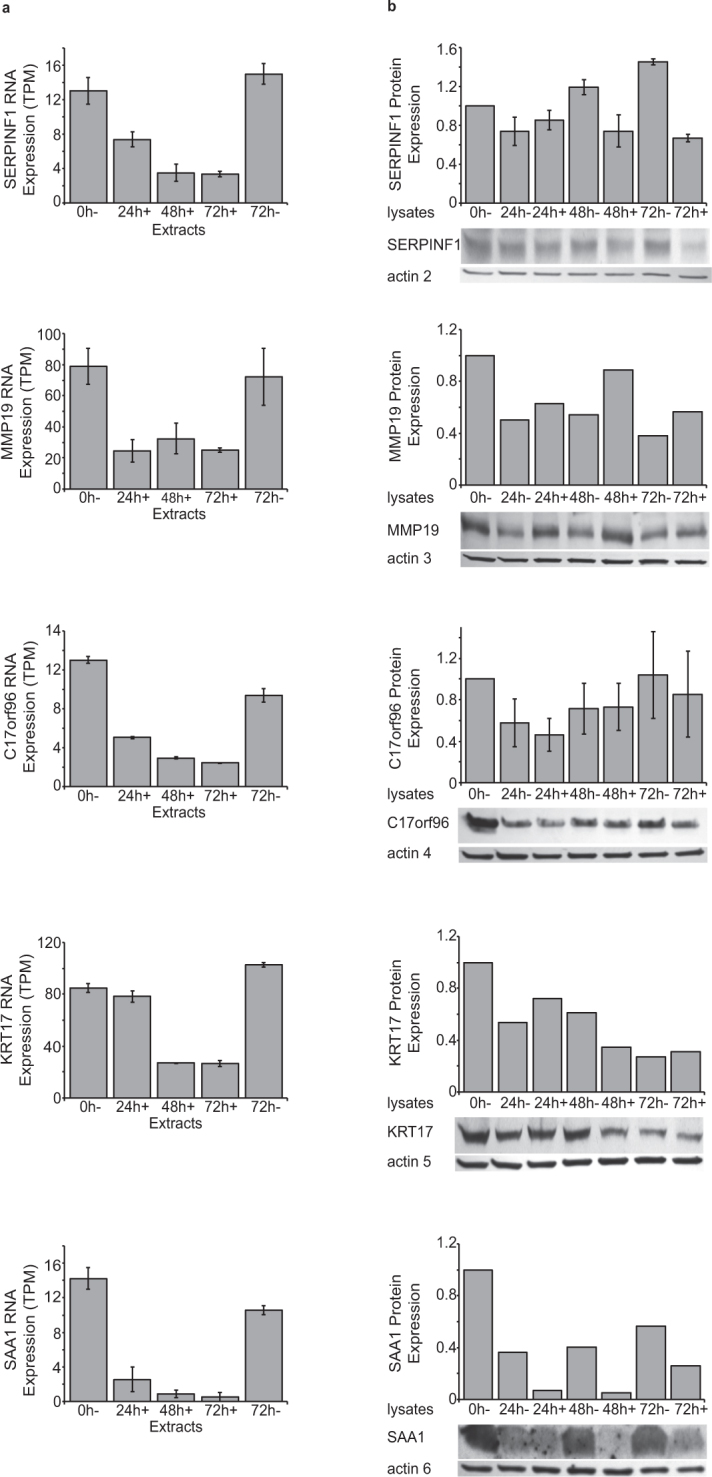
(**a**) RNA-Seq analysis of down-regulated genes of interest in vehicle treated BeWo cells (−) or BeWo cells treated with forskolin (+) for the times described in Materials and Methods. Results are expressed in transcripts per million (TPM). (**b**) Quantitative Western blot analysis of down-regulated genes of interest in vehicle treated BeWo cells (−) or BeWo cells treated with forskolin (+) for the times described in Materials and Methods. Immunoblots were quantified and normalized to levels of actin and subsequently to the 0h- sample of each blot.

Similarly, changes in the protein and RNA expression for the five genes which are down-regulated were seen in Fig. 7 and Table 1. The decrease in RNA expression was relatively small for four (SERPINF1, MMPI9, C17orf96, KRT17). In three of these genes (MMP19, C17orf96, KRT17) there was minimal change in protein expression. Although SERPINF1 showed a decrease in RNA expression similar to these three genes, SERPINF1 showed the greatest decrease in protein expression. Among the down-regulated genes, the decrease in RNA expression for SAA1 was the greatest. Although SAA1 showed a significant decrease in protein expression, the diminished expression was not as great as the decrease in SERPINF1. As with the overexpressed proteins, several of these proteins (SERPINF1, SAA1, KRT17 and MMP19) are secreted and thus, our measured levels of protein expression may not reflect the entire spectrum of protein expression by the cells. From these analyses, we conclude that RNA-Seq closely reflect changes in protein abundance in these two models of human cytotrophoblast transition to syncytiotrophoblast.

## Discussion

The data herein show a comprehensive, unbiased, RNA-Seq screen that identified genes which likely play a pivotal role in morphological and hormonal differentiation of cytotrophoblasts into syncytiotrophoblasts. The RNA-Seq analysis on samples from two complementary and biologically relevant *in vitro* model systems of syncytialization, including primary cultures of cytotrophoblasts transitioning to syncytiotrophoblasts from human term placentas derived from uncomplicated pregnancies, and forskolin-induced differentiation of cytotrophoblasts to syncytiotrophoblasts in the b30 clone of the human choriocarcinoma-derived cell line BeWo. We used stringent criteria to identify the most highly up-, and down-, regulated genes in the two model systems interrogated from the RNA-Seq data. We identified 11 genes from this analysis, with six up-regulated and five down-regulated, and pursued evaluation of protein expression of each of these in the BeWo system via quantitative Western blot analysis. The data show that the RNA and protein expression levels correlate closely, with the single exception being the expression of INHA. We speculate that this incongruence may reflect the fact that INHA is a secreted protein, and our analysis may therefore not reflect the actual expression of INHA.

Human chorionic gonadotropin is secreted by syncytiotrophoblasts and is a pivotal hormone for the maintenance of pregnancy from implantation until delivery. Gonadotrophic glycoproteins such as HCG are composed of two subunits, a common α subunit and a protein specific β subunit. Whereas, increased production of hCGβ has been used as a surrogate for syncytialization by several investigators^10,20,22^, others urge caution with this notion as syncytial fusion and hCGβ production may well be independent^23^. Our results with hCGβ are an excellent indication that both of our cell models of trophoblast transition from mononuclear to multinuclear trophoblast are valid models of syncytialization.

The five upregulated genes are either secreted proteins or receptors. Peptidylglycine α-amidating monooxygenase (PAM) is a bifunctional enzyme that catalyzes the amidation of bioactive peptides at the carboxy terminus to effect biological activity. Bioactive peptides include neurotransmitters, hormones, and paracrine signaling molecules^24^. Cysteine –rich intestinal protein 2 (CRIP2), a LIM domain protein and transcription regulator, expressed highly in the heart among numerous other tissues including the placenta, plays a role in development of each of these tissues^25^. Triggering receptor expressed on myeloid cells-like 2 (TRML2), also known as trem-like transcript 2 (TLT-2), is a cell surface receptor that binds CD276 on T-cells and plays a role in T-cell activation during innate and adaptive immunity^26^. Follistatin-related gene (FLRG), also known as follistatin-like-3(FSTL-3), is a product of a member of the follistatin gene family and thus, has similar structure and function to that of follistatin. Human placental trophoblasts synthesize this protein during pregnancy, and human trophoblasts up-regulate expression of both the gene and protein product in response to hypoxia^27^. Notably, FLRG (FSTL-3) binds activin with high affinity to modulate endothelial cell proliferation and angiogenesis^28^. Activin is also produced in the placenta and is a well-known component of pregnancy screening for risk assessment of aneuploidy. Similar to FLRG, inhibin alpha (INHA) also modulates the effects of activin, as an antagonist to activin receptor binding^29^. Like activin, INHA is a member of the TGFβ superfamily, is one of the subunits of TGFβ, is part of activin A and activin AB, and is produced in human placental trophoblast^30^. The fact that both FLRG and INHA are produced in the placenta suggests that their up regulation is a result of, rather than a requirement for, syncytial formation. As with hCGβ, the relationship between the expression of these two proteins and syncytial formation requires further study.

RNA and protein expression of the down-regulated genes was not as consistently coordinated as that seen with the up-regulated genes; however, the two genes whose RNA expression is most down-regulated (i.e., SAA1 and SERPINF1) also show the greatest reduction in protein expression. Elongin BC and Polycomb Repressive Complex 2-associated protein (EPOP, a.k.a. C17orf96) interact with multiple molecules to modulate chromatin configuration and to effect transcription in mammalian cells. C17orf96 generally up-regulates transcription, suggesting this factor is key in the regulation of long-term developmental processes^31^. Pigment epithelium-derived factor (PEDF, a.k.a. SERPINF1), a multifunctional, non-inhibitory member of the serine protease inhibitor (SERPIN) superfamily, is a potent neurotrophic factor acting in the eye and spinal cord, is a potent inhibitor of angiogenesis^32^, and acts as a tumor suppressor via binding to high affinity ligands and cell-surface receptors^33^. Serum amyloid A1(SAA1), a serum protein precursor involved in the formation of amyloid A, is involved in high density lipoprotein remodeling, and may play a role in the regulation of inflammation and immunity^34^. Matrix metalloproteinase 19 (MMP19) is a member of a group of endopeptidases that degrade components of the extracellular matrix during processes such as development and wound healing^35^. Keratin 17 (KTR17) is a type I keratin that participates in epidermal development and repair after tissue injury in mammals^36^, in part by promoting differentiation of selective epithelia^37^.

Taken together, we conclude that the activities of these eleven coordinately regulated gene products do not appear to behave as a single uniform function during the transition from mononucleated cytotrophoblast to differentiated syncytiotrophoblast. However, eight of the eleven proteins are secreted and are involved in cell signaling, either directly (INHA, hCGβ, FLRG, SERPINF1, SAA1) or indirectly by affecting signaling proteins or the extracellular matrix (PAM, MMP19, KRT17). Although the three remaining proteins are not secreted, two of them regulate transcription (CRIP2 and C17orf96) and one is a receptor (TREML2). The fact that all of these gene products are directly or indirectly involved in cell signaling remains consistent with the overall process of syncytial formation, which must involve cell-cell signaling given the nature of the fusion process.

Our RNA-Seq data was also examined for several other factors associated with cytotrophoblast fusion. Herein, both syncytin-1 and syncytin-2 were up-regulated in the presence of forskolin in BeWo cells whereas both were down-regulated in human placental syncytiotrophoblasts. By contrast, the transcript levels for the syncytin-1-receptor and syncytin-2 receptor, SLC1A5 and MFSD2A respectively, were largely static in vehicle-treated BeWo cells. Further, MFSD2A was expressed at very low levels (<1 TPM) at every time point regardless of forskolin treatment. In human placental samples, SLC1A5 was slightly up-regulated while MFSD2A was markedly up-regulated. Consistent with a critical role of GCMa^38^ in cyclic AMP-mediated syncytialization in BeWo cells, we observed an increase in GCMa transcript levels in the presence of forskolin that increased over time. However, there was effectively no change in the transcript levels of GCMa in human placental samples. TWIST1^39^ was not expressed in BeWo cells at any timepoint, regardless of forskolin treatment, yet was consistently expressed in human placental cells, but with no marked up- or down-regulation during syncytialization. ROCK1 and RAC1^40^ were expressed at similar levels across all time points and treatments. Both LAT1 and 4F2hc^41^ were upregulated 3–4 fold in forskolin-treated BeWo cells at 72 hours. In human placental cells, there was little change in transcript levels for these two genes. While we identified no change in Annexin-A5^42^ transcript levels in either BeWo cells or human placental cytotrophoblast cultures during the process of syncytialization, this gene was among the top 5% of transcripts at all timepoints. In addition, we observed high expression of PLAC1^43^ in syncytiotrophoblasts derived from human placental cells. This was the result of a greater than 5-fold up-regulation of this transcript from placental cytotrophoblasts. In contrast to the placenta samples, BeWo cells expressed only low levels at baseline, and down-regulated PLAC1 expression to less than one third of baseline with the addition of forskolin. Furthermore, there was no appreciable change in Galectin-1^11^ expression in either BeWo cells or human placental samples. Finally, our RNA-Seq did not detect IKCa^10^ transcripts at any time point in any of our samples.

Gene expression changes in BeWo cells^44–47^, term human placental samples^48^, or JEG-3 cells co-cultured with human microvascular endothelial cells^49^ have been reported by other investigators. Via microarray analysis, Kudo *et al*.^44^ examined the changes in gene expression during BeWo trophoblast differentiation. Zheng *et al*.^46^ performed RNA-Seq yielding data for BeWo cells of the parent choriocarcinoma cell line, to identify and cluster differentially expressed genes. Shankar and colleagues^45^ augmented their RNA-Seq data with DNA methylation and ChIP seq assays to generate a more complete epigenetic profile of BeWo cytotrophoblasts and syncytiotrophoblasts. Importantly, Rouault *et al*.^48^ employed microarrays to explore gene expression changes in villous cytotrophoblast and syncytiotrophoblasts in culture, and identified three transcriptional regulators (PPARγ, RARα, and NR2F1) of key importance to villous trophoblast differentiation. None of these three genes identified by Roualt *et al*.^48^ or of the thirteen genes identified by McConkey *et al*.^49^ were identified in our analysis of BeWo and human placental trophoblasts. McConkey *et al*.^49^ developed a novel method of syncytialization, by three dimensional co-culture of the choriocarcinoma JEG-3 line with human microvascular cells, in which JEG-3 cells form syncytia and assume gene expression changes similar to that seen in primary human syncytiotrophoblasts. In microarray analysis of BeWo cells + forskolin for 48 h, Renaud *et al*.^47^ identified OVOL1 as an up-regulated transcript and cognate protein and went on to demonstrate its role as a transcriptional regulator of trophoblast development. We also found OVOL1 up-regulated in BeWo + forskolin, however it was not up-regulated in the human placental cells and thus was not selected for further analysis. Our study is the first to use RNA-Seq to quantify gene expression in both primary human cytotrophoblasts and the syncytiotrophoblasts that evolve in culture. Moreover, we concurrently performed RNA-Seq on the BeWo b30 cell line, which was derived from the parent BeWo and which exhibits a marked capacity for syncytiotrophoblast formation in the presence of cyclic AMP stimulators. Comparing the commonalities and differences for the expressed genes between the two trophoblast phenotypes, and between our two models, allows us to better understand mediators that are pivotal in trophoblast syncytium formation. Moreover, our pursuit of changes in protein expression levels of differentially expressed genes buttresses the roles that these genes play in trophoblast differentiation.

## Materials and Methods

### Cells and Culture

BeWo b30 cells were cultured in F-12K Nutrient Mix, Kaighn’s Modification (Gibco), 10% FBS and 2 mM glutamine^20^. At 10% confluency, media was replaced with fresh media containing either 1% DMSO (control) or 100 µM forskolin in 1% DMSO at 0 h, 24 h, or 48 h as indicated. At the appropriate time, cells, media or both were harvested for RNA, protein analysis or immunofluorescence as indicated.

Human placental cytotrophoblasts were isolated from term human placentas of uncomplicated pregnancies. This study was approved by the Institutional Review Board of Washington University School of Medicine (IRB #201101889) which allowed verbal consent as no patient data was obtained other than the knowledge that the placenta was from a normal, term pregnancy. Informed consent was obtained from all study participants. All methods were performed in accordance with the relevant guidelines and regulations as previously described^50–52^. Following isolation, cells were plated at 3 × 10^5^ cells/cm^2^ and maintained in Dulbecco’s modified Eagle’s MEM containing 10% fetal calf serum, 20 mM HEPES pH 7.4, penicillin (100 u/ml), streptomycin (100 µg/ml) and fungizone (0.25 µg/ml) in 5% CO_2_/air. At 48 h, >85% of cells are multinucleated syncytiotrophoblast, as described earlier^53^. At the appropriate time (24 h for cytotrophoblasts, 72 h for syncytiotrophoblasts) cells were harvested for RNA or protein analysis as indicated.

Human placental villous extracts were prepared from term placentas as described^51^. Briefly, five, 5 mm^3^ samples of villous tissue were removed ~2 cm from the cord insertion but within the chorion frondosum, avoiding the basal plate. The samples were washed in PBSc, a phosphate buffered saline solution (DPBS, Cellgro) supplemented with 100 mM CaCl_2_ and 50 mM MgCl_2_ and homogenized in PBSc-containing 0.5% Igepal CA-630 (Sigma), Complete Protease Inhibitor Cocktail (Roche), and 1 mM DTT, for at least 30 min^54^ at 4 °C, centrifuged for 10 min at 13,000 × g at 4 °C and the supernatant (“lysate”) stored at −20 °C.

### Protein Analysis

For cellular protein analysis BeWo cells or human placental trophoblasts were rinsed in PBS at 4 °C and thereafter incubated for 20 min at 4 °C with fresh lysis buffer, followed by centrifugation (15 min at 13,000 × g at 4 °C) and the resultant supernatant was stored at −20 °C, as described previously^54^. Western blot analysis following SDS-PAGE was performed on aliquots and probed with antibodies as described in the text and Supplemental Table 6. Actin content was evaluated on each sample as a control and for normalization. Human placental villous extracts also served as a control. Western blots were developed with Pierce ECL Western Blotting Substrate or SuperSignal West Femto Maximum Sensitivity Substrate from Thermo Scientific and results were quantified as described previously^54^. Following normalization to actin, quantification of each protein was presented as amount relative to the corresponding zero-hour sample. In general, 2–3 independent analyses were performed for each protein.

Media samples from BeWo cells were analyzed directly via Western blot as described previously^20^. Selected samples of cell lysate or media were treated directly with De-glycosylation Mix II from New England Biolabs under denaturing conditions.

### Immunofluorescence Analysis

For immunofluorescence analysis and as described previously^20,54^ BeWo cells were seeded onto glass coverslips in six well dishes and treated with forskolin or DMSO as described above. Cells were then washed with PBSc, fixed with 4% paraformaldehyde and permeabilized in 1% Triton-X 100. After blocking in 1% BSA/PBSc, coverslips were immunostained with primary antibody to determine subcellular localization. After washing with PBSc, coverslips were incubated with the appropriate AlexaFluor 488 or 568 secondary antibodies (Invitrogen/Molecular Probes) and DAPI and mounted using Mowiol containing 2.5% 1, 4-diazo-bicyclo-[2.2.2]-octane (DABCO, Sigma). Images were captured using an Olympus FluoView 1000 confocal laser scanning microscope, and Olympus FV10-ASW software, Version 3.0.

### Immunohistochemistry Analysis

For immunohistochemistry analysis, human placental villous tissue was harvested as described above, washed in PBS_c_, fixed in 4% paraformaldehyde and embedded in paraffin. Tissue sections were processed via standard histological techniques and immunostained with antibodies listed in Supplementary Table 6 followed by HRP-secondary antibodies, incubation with HRP substrate and H and E counterstain. Images were captured using a Zeiss Axioskop microscope with Zen software.

### RNA-Seq

Total RNA was isolated from BeWo and human trophoblasts using the RNeasy Plus Mini Kit (Qiagen) according to the manufacturer’s instructions. QIAshredder was used for homogenization. The amount of total RNA extracted was quantified on a Nanodrop 2000 spectrophotometer and RNA quality and purity was further evaluated using an Aligent 2100 Bioanalyzer (Aligent Technologies, Santa Clara, CA, US). RNA-Seq was performed at the Genome Technology Access Center in the Department of Genetics at Washington University School of Medicine. Briefly, ribo-depleted libraries were prepared from the RNA, and samples were indexed and sequenced on an Illumina HiSeq. 2500. Sequencing results from this study have been deposited in the NCBI Short Read Archive under accession number PRJNA397241.

Raw reads were pseudo-aligned using kallisto version 0.42.4 using options “—single –b 10 –l 220 –s 20”^55^. Transcript abundance and initial differential expression analysis was performed using the sleuth package^56^ in R version 3.2.1.

### Bioinformatic Analysis

All analyses were performed in R version 3.2.1. Transcript abundance was determined by sleuth and measured in transcripts per million (TPM). To determine which transcripts are differentially expressed, we applied a filter requiring a transcript to be expressed at or above the 85th percentile in at least one sample, and have a 2.5-fold change in expression between syncytiotrophoblast samples and cytotrophoblast samples. Parallel analyses to identify differentially expressed genes was performed as follows. Reads were aligned to the hg19 reference genome using STAR^57^. The featureCounts method of the subread^58^ package was used to determine the number of reads that aligned to each gene. Finally, differential gene expression was determined by comparing 72 + and 72− BeWo samples and comparing 24 h and 72 h primary placental samples using the EdgeR package in R^59^ and the Cuffdiff package^60^. To generate the heat maps, one sample was designated as the index and used to sort the transcripts. The transcript levels for every other sample were ordered according to this index sample and displayed as log(TPM).

Gene list enrichment was performed using the Panther^21^ online suite of tools. We provided a list of the most significant genes as input. The resulting gene ontology categories were reported. The default setting of the Panther analysis is to perform a Bonferroni correction for multiple test correction. Thus, the reported results are unlikely to be false positives.

### Data Availability Statement

The sequencing results from this study have been deposited in the NCBI Short Read Archive under accession number PRJNA397241.

## Electronic supplementary material

Supplementary Tables 1, 2, 3, 6 + Figures 1, 2, 3

Supplementary Table 4

Supplementary Table 5
