# Supplementary material for: RNA-Seq identifies genes whose proteins are transformative in the differentiation of cytotrophoblast to syncytiotrophoblast, in human primary villous and BeWo trophoblasts
Source: Sci Rep. 2018 Mar 23;8:5142. doi: 10.1038/s41598-018-23379-2 (PMC5865118; doi:10.1038/s41598-018-23379-2)
Supplement: Supplementary file 1 — Supplementary Tables 1, 2, 3, 6 + Figures 1, 2, 3 [file 41598_2018_23379_MOESM1_ESM.pdf]

RNA-Seq identifies genes whose proteins are transformative in the differentiation of cytotrophoblast to syncytiotrophoblast, in human primary villous and BeWo trophoblasts

Christopher Azar[1#], Mark Valentine[2#], Julie Trausch-Azar[1], Todd Druley[1,2], D. Michael Nelson[3], and Alan L. Schwartz[1,4*]

Departments of Pediatrics[1], Genetics[2], Obstetrics and Gynecology[3] and Developmental Biology[4], Washington University School of Medicine, St. Louis, MO 63110. USA.

[#]Co-first authors

*Correspondence to A.L.S.: Schwartz@wustl.edu

Supplemental Table 1: The 50 most up-regulated or down-regulated genes in BeWo syncytiotrophoblasts compared to cytotrophoblasts.

Transcript	Gene	Fold Change 72- to 72+	Fold Change pc to ps
ENST00000453881	THEM5	103.8472782	1.570353653
ENST00000357383	CGB	47.28902292	10.47140062
ENST00000248553	HSPB1	44.85826992	1.173231331
ENST00000301408	CGB5	43.88428825	11.20380374
ENST00000435414	TFPI	42.94830801	0.878900571
ENST00000448456	CGB8	41.5608602	7.368405229
ENST00000377280	CGB7	37.9799357	6.213483916
ENST00000533259	PDE4DIP	37.26509984	1.038996747
ENST00000449864	HDLBP	36.9507669	0.686891204
ENST00000483722	TREML2	36.28412543	3.408048567
ENST00000369582	CGA	34.92592109	5.052257188
ENST00000315491	TUBB3	34.83337686	0.485880951
ENST00000447124	SDC1	33.90614584	1.592858209
ENST00000489921	TNFRSF1B	33.28498413	0.062651649
ENST00000617224	FAM25C	33.27984808	0.200312921
ENST00000594480	LGALS16	32.56444868	0.607238551
ENST00000441322	CKMT1B	31.60528365	2.370200794
ENST00000441322	CKMT1A	31.60528365	2.370200794
ENST00000393062	SPIRE2	31.01552906	1.580708729
ENST00000528858	ST3GAL4	29.83010339	0.479050341
ENST00000553379	HOPX	29.58126719	1.461676623
ENST00000243786	INHA	28.89794279	5.52952087
ENST00000328965	OAF	28.34985073	0.704864514
ENST00000301335	SLC43A2	27.49413108	0.842228303
ENST00000429147	SLC6A8	25.45752726	0.970979807
ENST00000400920	PRKCZ	25.19596569	0.643696571
ENST00000518010	NDRG1	25.1824057	2.002908743
ENST00000542313	ULK1	25.15382818	0.628626416
ENST00000396650	C15orf48	22.25767832	0.722664266
ENST00000589695	TREM1	22.22858073	1.623220483
ENST00000613488	LOC102724428	22.21052429	0.405379128
ENST00000435224	ABTB2	20.57306522	0.80943686
ENST00000626960	BCAS3	20.01650533	2.328928252
ENST00000602168	ERVV-1	19.1092545	0.380570701
ENST00000489808	EPHB4	18.93924132	1.706615436
ENST00000244333	LYPD3	18.80414315	0.246198288
ENST00000333007	TNFAIP2	18.50784447	0.793115265
ENST00000493893	COMT	18.34659265	1.697889817
ENST00000245105	SH3TC1	18.08188124	0.972860434
ENST00000369583	DUSP5	17.89983622	0.470499162
ENST00000342784	SYDE1	17.87112424	1.282100091
ENST00000443742	SLC19A1	17.74043273	1.380087721
ENST00000609656	FKBP8	17.19829716	0.83554995
ENST00000507502	RAI14	17.15147202	0.689911536
ENST00000542629	PPFIBP1	16.96125455	0.629971172
ENST00000420781	FNBP1	16.82534652	0.557390058
ENST00000553143	TCP11L2	16.40843733	1.004335037
ENST00000331569	ZNF703	16.34893843	1.346921808
ENST00000511996	AFF1	16.25111219	0.71994096
ENST00000494726	GADD45G	15.93770234	0.730371381
ENST00000462208	OVOL2	0.015934018	2.313746495
ENST00000445236	ACHE	0.03500923	0.549546585
ENST00000611954	IFI27	0.062669965	0.757551831
ENST00000542056	GPRC5A	0.075469952	1.219882044
ENST00000357001	GUCA2A	0.078803028	0.465948314
ENST00000376468	NPPB	0.078837749	0.940321261
ENST00000627248	RIN1	0.079754861	0.944332445
ENST00000255688	RARRES3	0.080926213	1.851810474
ENST00000466829	COL16A1	0.082085867	0.629714559
ENST00000409652	APOL6	0.103544509	1.186647614
ENST00000372980	JPH2	0.117681572	0.97881129
ENST00000337554	TSPO	0.120770862	1.953013983
ENST00000331289	ASCL2	0.131332955	1.884785652
ENST00000464047	NMNAT2	0.136882532	1.100894229
ENST00000319560	TMEM31	0.145442303	0.689708737
ENST00000475296	PLS1	0.146319143	1.035204945
ENST00000518884	LY6D	0.149208093	2.745917561
ENST00000217289	FERMT1	0.151474539	1.011083212
ENST00000345366	CSH2	0.154153825	4.753002086
ENST00000601760	SLC25A23	0.154830527	0.657469419
ENST00000494431	SYT8	0.163657905	0.780472213
ENST00000585928	WTIP	0.164644107	2.410481243
ENST00000558028	TGIF2	0.167983551	1.594559507
ENST00000409223	KLHL30	0.173189229	2.161848438
ENST00000537006	ENC1	0.174770306	0.975523818
ENST00000473723	SUN3	0.176288766	1.544713439
ENST00000329305	TPM2	0.176552966	2.386586482
ENST00000257435	NREP	0.181131968	1.000931875
ENST00000380345	LHFPL2	0.186829144	1.054914319
ENST00000370473	GBP1	0.18912621	0.617551021
ENST00000467054	DBN1	0.191735056	1.584119062
ENST00000478217	F3	0.19320273	1.381102749
ENST00000374882	PSMB8	0.19468191	1.127599176
ENST00000456543	GH2	0.197619827	16.87691281
ENST00000426972	DDAH1	0.19945478	0.722514011
ENST00000370466	GBP2	0.199789665	0.680404733
ENST00000303921	GPR37	0.201063032	1.290277219
ENST00000344366	CA12	0.202386701	1.447633124
ENST00000486622	RNF144B	0.204018718	1.188574161
ENST00000261402	NUAK1	0.204554702	1.171539264
ENST00000235345	SLC35D1	0.205398449	1.200481884
ENST00000161006	PRSS22	0.206521669	1.060689828
ENST00000557872	CHRM5	0.211674341	1.315731937
ENST00000520886	ANKRD6	0.213078576	3.669853811
ENST00000532858	SAA1	0.216613087	0.390191959
ENST00000264025	PVRL1	0.217103438	1.448759128
ENST00000359237	PLAC1	0.221123081	5.2752825
ENST00000585544	NFKBID	0.224181066	1.160226529
ENST00000532150	WNT11	0.227985391	2.381232682
ENST00000581694	GATA6	0.229438918	1.20924383

Supplemental Table 2: The 50 most up-regulated or down-regulated genes in term placental syncytiotrophoblasts compared to cytotrophoblasts.

Transcript	Gene	Fold Change 72- to 72+	Fold Change pc to ps
ENST00000456543	GH2	0.197619827	16.87691281
ENST00000301408	CGB5	43.88428825	11.20380374
ENST00000357383	CGB	47.28902292	10.47140062
ENST00000625357	KISS1	0.876643514	7.721236937
ENST00000558670	GCHFR	0.506507223	7.559892269
ENST00000509646	XPNPEP1	2.290619725	7.389544232
ENST00000448456	CGB8	41.5608602	7.368405229
ENST00000518173	PTK2	2.109548072	6.887995498
ENST00000625685	ABR	4.354473462	6.623854321
ENST00000377280	CGB7	37.9799357	6.213483916
ENST00000592535	APOC1	0.650247642	6.151633599
ENST00000525804	ANO9	1.394214764	5.882865527
ENST00000475053	NT5C3B	1.28151847	5.719605253
ENST00000243786	INHA	28.89794279	5.52952087
ENST00000558074	TGM1	0.609371321	5.487162367
ENST00000559745	FAM63B	2.482934143	5.448002649
ENST00000359237	PLAC1	0.221123081	5.2752825
ENST00000549106	ALDH2	0.303096925	5.264868742
ENST00000534526	FGD4	0.699896364	5.206122174
ENST00000248121	SYNGR3	0.724736137	5.144651034
ENST00000369582	CGA	34.92592109	5.052257188
ENST00000466020	CLHC1	1.545204309	4.984602441
ENST00000626005	OXSM	2.10223063	4.950340733
ENST00000318059	PAM16	1.801081518	4.922355403
ENST00000239316	INSL4	13.57641886	4.912297625
ENST00000492324	VDAC1	1.319450097	4.851983691
ENST00000425781	TEX264	1.474671736	4.845961307
ENST00000415028	HYAL2	1.035312377	4.767821365
ENST00000345366	CSH2	0.154153825	4.753002086
ENST00000316193	CSH1	0.239546064	4.732145957
ENST00000316193	CSHL1	0.239546064	4.732145957
ENST00000406487	PSG2	1.089349583	4.663489904
ENST00000585390	ST6GALNAC2	1.36414233	4.507116785
ENST00000467667	TFDP2	0.845796353	4.393735085
ENST00000354900	LSR	3.543223288	4.354658896
ENST00000429040	SLC39A1	4.270715665	4.342823138
ENST00000392462	MORN3	0.791513381	4.341158972
ENST00000433032	TFEB	1.739106593	4.338417424
ENST00000488982	ARMCX2	1.268477764	4.234993074
ENST00000494106	GALNT2	9.764932545	4.201982284
ENST00000538259	CRIP2	9.213179505	4.17300502
ENST00000479079	PIK3C2B	3.572749447	4.119126613
ENST00000369462	BRCC3	1.886955086	4.101319654
ENST00000583961	GPS1	2.472339454	4.096512947
ENST00000529384	ARL2	1.335439961	4.092213458
ENST00000341184	MGAT3	0.873982927	4.086488361
ENST00000466494	IDH3B	1.148947935	3.967157702
ENST00000452853	PFN2	1.294019783	3.964744852
ENST00000375077	CORO2A	2.008152979	3.964086045
ENST00000254193	SNRPA1	8.624439181	3.945532817
ENST00000368723	S100A7	3.436868952	0.034598968
ENST00000520511	FBXO32	5.132259504	0.045899495
ENST00000489921	TNFRSF1B	33.28498413	0.062651649
ENST00000472091	ERVFRD-1	8.409083095	0.071896586
ENST00000514755	UBE2D3	3.052654193	0.072316819
ENST00000367054	SOD2	4.978863174	0.083624093
ENST00000367054	LOC10012951[illegible]	4.978863174	0.083624093
ENST00000569500	MT1G	0.869903889	0.090169041
ENST00000368738	S100A9	0.80869126	0.107569917
ENST00000332374	MT1H	0.792164554	0.118039972
ENST00000428570	SLC22A11	1.930486005	0.145160274
ENST00000628955	PSMA6	2.896561968	0.148526521
ENST00000295453	ALPPL2	3.222998492	0.1579152
ENST00000306061	MT1E	1.024725459	0.164803879
ENST00000461655	PPAP2B	3.448108226	0.166603462
ENST00000497822	C1orf61	0.498153467	0.173824516
ENST00000563985	MT2A	0.820973788	0.1863297
ENST00000394485	MT1X	0.592207615	0.190279045
ENST00000589593	FOSB	10.96291913	0.196210327
ENST00000515463	SRP19	2.443895545	0.197157041
ENST00000569036	CDH3	0.552515391	0.198995591
ENST00000617224	FAM25C	33.27984808	0.200312921
ENST00000614327	HSP90B1	12.87924205	0.218323671
ENST00000457104	VEGFA	1.048150001	0.22073577
ENST00000480376	SLC13A4	11.60653778	0.223468137
ENST00000519760	CDK1	0.818064976	0.226276426
ENST00000613024	C17orf96	0.260643882	0.231650999
ENST00000252115	POLDIP3	2.653922739	0.232320274
ENST00000366555	KMO	1.505177155	0.236862091
ENST00000182377	FAR2	6.840339493	0.237950361
ENST00000430989	SUCLG1	1.77122875	0.241585905
ENST00000519287	PTTG1	0.764675768	0.242081878
ENST00000235382	RGS2	2.945296557	0.244361135
ENST00000244333	LYPD3	18.80414315	0.246198288
ENST00000464451	SEC61A1	6.577310205	0.246418502
ENST00000329402	GAST	4.367427176	0.246843766
ENST00000373087	ZC3H12A	1.351693674	0.251241636
ENST00000418714	SLC40A1	10.68699937	0.253907114
ENST00000601417	ERVV-2	13.84765423	0.255536231
ENST00000488574	PEG10	3.722751015	0.257940145
ENST00000237305	SGK1	12.91441365	0.260736685
ENST00000577817	KRT17	0.308396334	0.262343417
ENST00000451952	TMEM198	1.796712779	0.269811629
ENST00000366981	ATF3	8.516293693	0.26996512
ENST00000547299	MMP19	0.349870698	0.270651981
ENST00000290551	BTG2	0.475593158	0.272181783
ENST00000469609	CEP85	3.684395609	0.274339838
ENST00000486444	NFKBIZ	5.002917615	0.276703977
ENST00000546917	NACA	2.513036631	0.276712934
ENST00000559656	DET1	4.752363972	0.277484241

Supplemental Table 3: Summary of Panther Pathway analysis. The 250 most significantly differentially expressed (DE) genes were provided as inputs to PANTHER. The Gene Ontology biological processes that were enriched for our list of differentially expressed genes are listed.

GO biological process	Number of Genes in Category	Number of DE Genes	Expected Number of DE Genes	Fold Enrichment	P-Value
amino acid transmembrane transport (GO:0003333)	70	8	0.69	11.63	5.09E-03
female sex differentiation (GO:0046660)	104	9	1.02	8.81	9.86E-03
positive regulation of angiogenesis (GO:0045766)	125	9	1.23	7.33	4.33E-02
positive regulation of vasculature development (GO:1904018)	142	10	1.39	7.17	1.56E-02
gonad development (GO:0008406)	210	13	2.06	6.3	1.88E-03
development of primary sexual characteristics (GO:0045137)	215	13	2.11	6.16	2.45E-03
sex differentiation (GO:0007548)	264	15	2.59	5.78	6.49E-04
reproductive structure development (GO:0048608)	416	23	4.09	5.63	3.18E-07
regulation of angiogenesis (GO:0045765)	219	12	2.15	5.58	1.94E-02
reproductive system development (GO:0061458)	420	23	4.13	5.58	3.83E-07
in utero embryonic development (GO:0001701)	321	14	3.15	4.44	3.82E-02
regulation of body fluid levels (GO:0050878)	495	21	4.86	4.32	2.31E-04
developmental process involved in reproduction (GO:0003006)	626	25	6.15	4.07	3.31E-05
blood vessel development (GO:0001568)	457	18	4.49	4.01	6.85E-03
vasculature development (GO:0001944)	479	18	4.71	3.83	1.32E-02
cardiovascular system development (GO:0072358)	488	18	4.79	3.76	1.71E-02
wound healing (GO:0042060)	465	17	4.57	3.72	3.74E-02
tube development (GO:0035295)	560	19	5.5	3.45	2.93E-02
anatomical structure formation involved in morphogenesis (GO:0048646)	824	24	8.09	2.97	2.03E-02
regulation of anatomical structure morphogenesis (GO:0022603)	952	26	9.35	2.78	2.40E-02
positive regulation of developmental process (GO:0051094)	1203	31	11.82	2.62	8.13E-03
reproductive process (GO:0022414)	1342	34	13.18	2.58	3.31E-03
reproduction (GO:0000003)	1343	34	13.19	2.58	3.36E-03
single organism reproductive process (GO:0044702)	1206	30	11.85	2.53	2.52E-02
regulation of apoptotic process (GO:0042981)	1420	35	13.95	2.51	4.12E-03
regulation of programmed cell death (GO:0043067)	1434	35	14.09	2.48	5.16E-03
regulation of cell death (GO:0010941)	1533	36	15.06	2.39	8.56E-03
regulation of multicellular organismal development (GO:2000026)	1734	39	17.03	2.29	8.13E-03
tissue development (GO:0009888)	1647	37	16.18	2.29	1.67E-02
regulation of cell proliferation (GO:0042127)	1560	35	15.32	2.28	3.42E-02
anatomical structure morphogenesis (GO:0009653)	1952	41	19.17	2.14	2.28E-02
regulation of developmental process (GO:0050793)	2279	47	22.39	2.1	5.85E-03
regulation of multicellular organismal process (GO:0051239)	2679	53	26.31	2.01	3.35E-03
cellular response to chemical stimulus (GO:0070887)	2389	47	23.47	2	2.17E-02
system development (GO:0048731)	4157	81	40.83	1.98	8.25E-07
animal organ development (GO:0048513)	2967	56	29.14	1.92	6.45E-03
response to organic substance (GO:0010033)	2605	49	25.59	1.91	4.40E-02
multicellular organism development (GO:0007275)	4746	87	46.62	1.87	2.63E-06
regulation of cell communication (GO:0010646)	3082	56	30.27	1.85	2.15E-02
regulation of signaling (GO:0023051)	3134	56	30.78	1.82	3.60E-02
anatomical structure development (GO:0048856)	5073	89	49.83	1.79	1.44E-05
positive regulation of cellular process (GO:0048522)	4889	85	48.02	1.77	7.12E-05
single-multicellular organism process (GO:0044707)	5527	96	54.29	1.77	3.09E-06
positive regulation of biological process (GO:0048518)	5437	94	53.41	1.76	7.32E-06
regulation of response to stimulus (GO:0048583)	3756	64	36.89	1.73	2.78E-02
single-organism developmental process (GO:0044767)	5349	90	52.54	1.71	9.58E-05
developmental process (GO:0032502)	5433	90	53.37	1.69	2.12E-04
multicellular organismal process (GO:0032501)	6615	102	64.98	1.57	5.22E-04
single-organism cellular process (GO:0044763)	9830	136	96.56	1.41	2.02E-04
single-organism process (GO:0044699)	12677	165	124.52	1.33	1.07E-05

Supplemental Table 6.

Protein	MW	Company	Antibody	Host	Lot #
hCGβ	18	Abcam	ab3976	Rabbit p	GR254346-B
PAM	108	Santa Cruz	sc-514110	Mouse m	L0214
CRIP2	24	Fisher	PA5-30997	Rabbit p	RF2225662E
TREML2/TLT2	37	Fisher	PA5-47471	Goat p	RF2225929
FLRG/FSTL3	27	USBio	035783	Rabbit p	L16102066
INHA	40	Abcam	ab81322	Rabbit m	YK022203DS
SERPINF1/PEDF	46	LS Bio	LS-B2505	Rabbit p	76652
MMP19	57	Fisher	PA5-28213	Rabbit p	RK2294709A
C17orf96/EPOP	50	LS Bio	LS-C20652	Rabbit p	76654
SAA1	13	Abcam	ab687	Mouse m	GR167376-9
KRT17	48	Fisher	PA5-27949	Rabbit p	RK2294708A
actin	50	Sigma	A5441	Mouse m	064M4789V

Description of antibodies used for immunoblots and immunohistochemistry of up-regulated and down-regulated genes. In host column p = polyclonal, m = monoclonal.

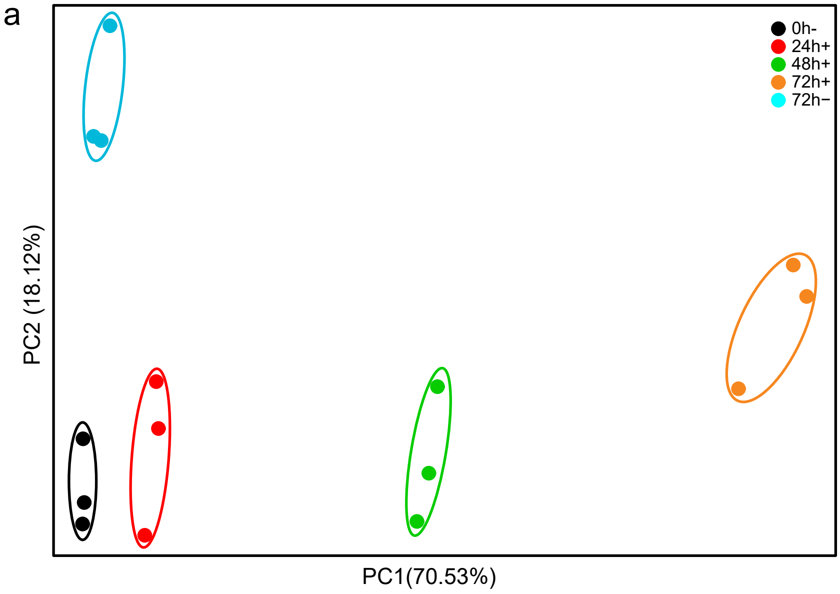

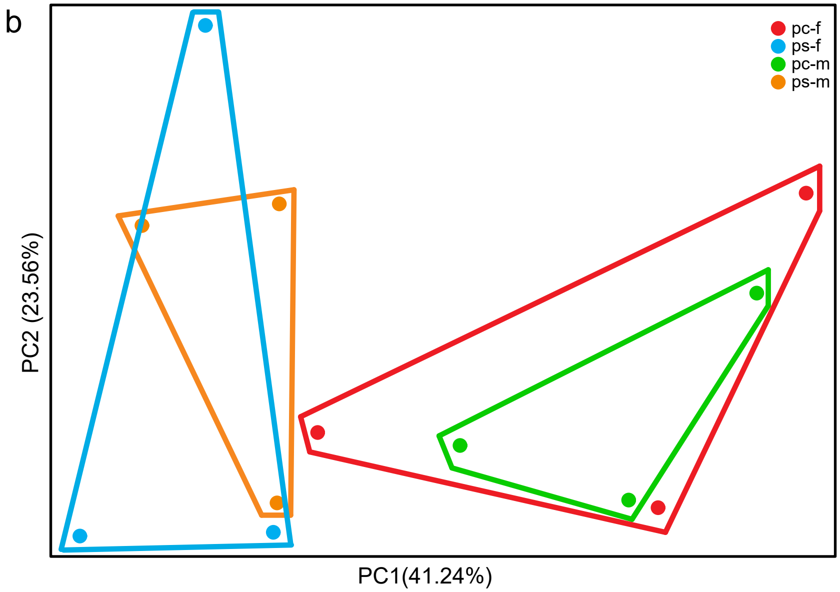

Supplemental Figure 1. Principle components analysis of BeWo cell (a) and placental trophoblasts (b) gene expression data was performed in R. The first and second principal components are plotted on the x- and y-axis respectively. Note: m=male placental samples and f=female placental samples.

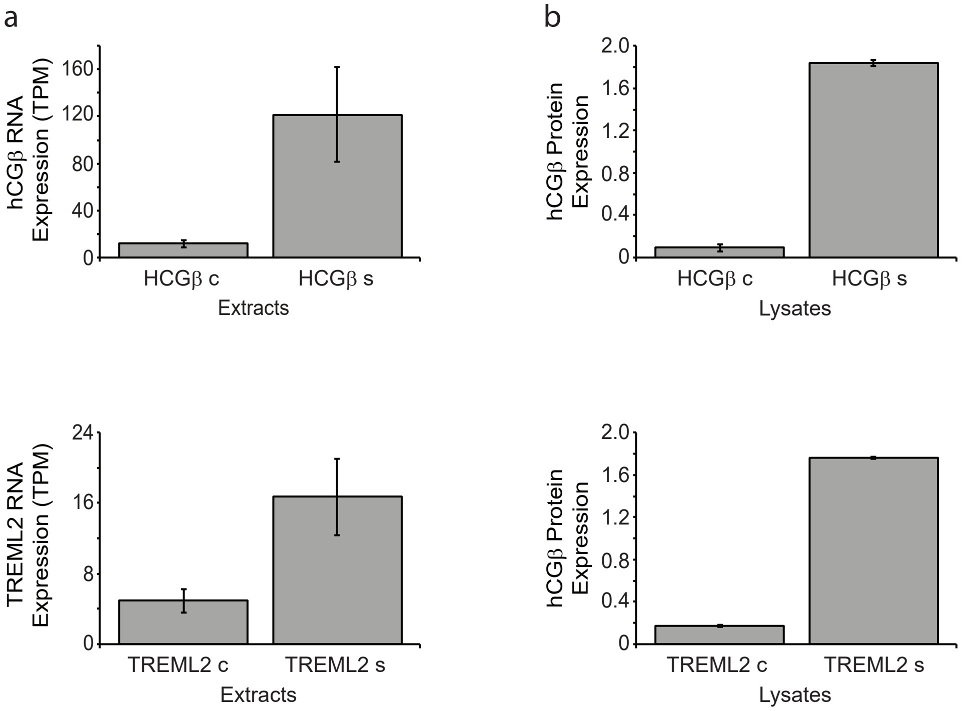

Supplemental Figure 2. a: RNA-Seq analysis of HCGβ and TREML2 in normal term human placental cytotrophoblasts (c) and syncytiotrophoblasts (s). RNA was isolated as described in Materials and Methods. Results are expressed in transcripts per million (TPM). b: Quantitative Western blot analysis of HCGβ and TREML2 in normal term human placental cytotrophoblasts (c) and syncytiotrophoblasts (s). Lysates were made from cells as described in Materials and Methods. Immunoblots were quantified and normalized to levels of actin in the lysates.

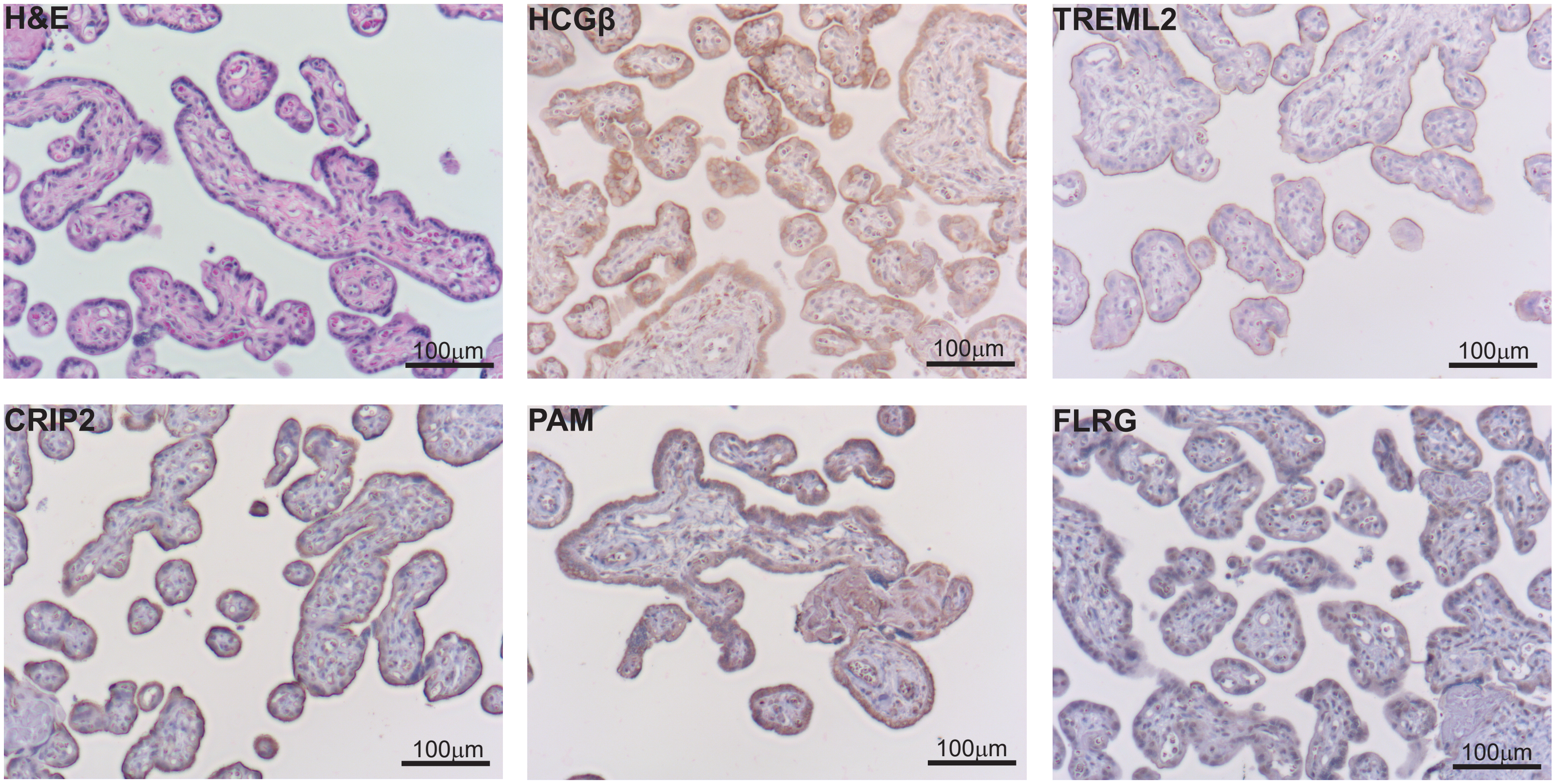

Supplemental Figure 3. Immunochemical staining of normal term human placental villi. Normal term human placental villi samples immunostained with antibodies to up-regulated proteins or hematoxylin & eosin as indicated. Robust HRP staining of syncytiotrophoblasts is clearly visible for up-regulated proteins. Note: TREML2, a membrane protein, shows very distinct plasma membrane staining.
